# Centralisation versus Decentralisation: How to Fight Tuberculosis

**Published:** 1917-06-16

**Authors:** 


### CENTRALISATION *versus* DECENTRALISATION.

### How to Fight Tuberculosis.

SELF-PRAISE is no recommendation, and much akin to self-praise is praise of those with no other claim to merit than that their opinion happens to agree with one's own. But if such ally is a person with already established claims to wisdom, then it is permissible to make something of the fact of his name and fame. We have aforetimes criticised the anti-tuberculosis campaign adversely, for faults and abuses in the actual working of the same, tracing these faults and abuses, in so far as avoidable, ultimately to the unfitness of the many local executives all over the country in charge of anti-tuberculosis measures. And in recommending centralised control, we have fortified ourselves by quoting Sir William Osler to the same effect. In his recent address before the Medical Society of London, on a kindred subject—namely, the campaign against syphilis—the Oxford Professor of Medicine has again made pointed remarks on this subject; as for instance:

"Recognising the existence of the disease [syphilis] as a great menace to public health, legislation has been enacted to fight the enemy on a settled plan at many centres under the control of the Local Government Board. It is a new departure to deal with an individual disease in this way.

"What a change a single word may effect! Tuberculosis, the white plague, is really a more hopeful disease to fight than syphilis. Though it has the same strong allies, poverty and drink, there is absent the complicating problem of prostitution. The word 'may' instead of 'shall' in the Tuberculosis Act gave us an ineffective guerilla warfare of local bodies for a Kitchener and a General Staff. The Government made no mistake this time—*Shall* is the word, and all over the country the clinics are in course of formation. Nurtured upon the Reports of the Local Government Board, I dwelt with a purpose on the successful campaign which it had waged against typhoid fever. In that great warfare Sir John Simon was chief of staff, and the battle was won by able lieutenants who directed the actual fighting in various parts of the country."

And again:

"Between the clinical and the laboratory side there will be enough at each clinic to occupy a large part of the time of a male and female doctor, who will, I trust, become the skilled advisers of the profession and of the public in each district. It should be our business to make these positions sufficiently attractive to catch the very best, and I am sure the hospital authorities will welcome them warmly as members on the staff. In large cities they might well be whole-time positions, though I should prefer to allow their colleagues and the public to have the benefit of their ever-increasing experience. . . . The clinic should be the centre in each district of an active educational propaganda which should be stimulated and planned by the general staff, and not left to the timid discretion of the local authorities."

Well, THE HOSPITAL may claim to have independently given details in proof, in advance proof as it were, of all this. At the beginning of this year, under the title "Reform in Tuberculosis Work," we published from a correspondent information as to the almost kaleidoscopic nature of the change in the medical superintendentship of public sanatoria, as to the insufficient prestige allowed the occupants of these posts, as to the general unfairness of elections to the position of tuberculosis officer, with consequent low clinical capability of these officers. At various times, too, we have pointed out the obvious fact that the range of the activities of a local medical officer of health is now far beyond his capacity. The report of a fresh instance of lack of success comes to hand only too conveniently, however, to be omitted. Here is a small county borough which wants its own anti-tuberculosis unit according to its own ideas. The scheme is drawn up by its own servant the medical officer of health, and an old house is bought and huts put up in the grounds. Mistake No. 1, for patients sleeping in huts may scamper off anywhere at night, and no one be the wiser. The huts face wrongly, some of them fill with snow in winter, and have to be closed. Tiny quarters and a small salary are all that can be offered for the combined post of sanatorium resident and tuberculosis officer. The cost of the scheme is said to have been under-estimated by the Town Clerk, and the working of it to be pinched in consequence. Result, three occupants of the post in three years, two of whom were without any proper special experience. All when leaving make no secret to their friends of dissatisfaction with the conditions, especially with the want of freedom. This is no way in which to fight the tremendously powerful bacillus of Koch, now beaten back to his inner defences, it is true, but stronger there, far stronger, than the majority thinks.

The only alternative is what Sir William Osler indicates. There may, indeed will, if it is adopted, be mistakes and abuses in that plan too, but they cannot be such bad ones. There can be no half-way house, such as giving the medical faculties of provincial universities charge of arrangements. That plan was essayed in more than one instance, and given up through opposition by the local municipality. Tuberculosis officers must possess certain attributes. They must not be young gentlemen fresh from any university, but must have lived long enough to have seen the failure of a few of the fifty million unsuccessful remedies yet tried for tuberculosis. They must have a diploma of public health, must have done six months post-graduate work at laryngology, and three months at least at Berck-sur-Mer or Alton. They must first of all have done resident work at a sanatorium. In sum, they must be tuberculosis experts, of whom the country does not own half a dozen at present.

The real difficulty is to find the men.

